# Age-Related Changes in Para and Wheelchair Racing Athlete’s Performances

**DOI:** 10.3389/fphys.2019.00256

**Published:** 2019-03-19

**Authors:** Julien Schipman, Pasquale Gallo, Andy Marc, Juliana Antero, Jean-François Toussaint, Adrien Sedeaud, Adrien Marck

**Affiliations:** ^1^Institut de Recherche Bio-Médicale et d’Épidémiologie du Sport, EA 7329, Institut National du Sport, de l’Expertise et de la Performance, Université Paris Descartes, Université Sorbonne Paris Cité, Paris, France; ^2^Institut de Formation en Masso-Kinésithérapie Valentin Haüy, Paris, France; ^3^Centre d’Investigations en Médecine du Sport, Hôtel-Dieu, Assistance Publique – Hôpitaux de Paris, Paris, France; ^4^Programme Interdisciplinaire de Recherche la Dynamique du Vieillir, Université Sorbonne Paris Cité, Paris, France; ^5^Laboratoire Matière et Systèmes Complexes, UMR 7057 CNRS, Université Paris Diderot, Université Sorbonne Paris Cité, Paris, France

**Keywords:** peak age, impairment and disability, track and field, paralympic games, classification

## Abstract

During the last decades, all para-athletes with disabilities have significantly increased their performance level due to technological progress and human investment, through better training or recovery protocols, medical care and nutritional monitoring. Among these elements, the athlete’s age is one of the determining factors in performance. The aim of this study was to investigate the effect of age on maximal performances for para-athletes and wheelchair racing athletes, scaled on able-bodied records. We collected 53,554 results including athlete’s best performance of the year, event, age and disability classification from the International Paralympic Committee competitions between 2009 and 2017 for both female and male para-athletics and wheelchair racing disciplines for a total of 472 sport events in Track and Field (considering each impairment type for each event) and gathered the all-time able-bodied records from the International Association of Athletics Federations (IAAF) at the end of 2017. Maximal performance by age was fitted with the Moore function for each para-athletics and wheelchair racing event. This study finds a similar age-related pattern in maximal performance among para-athletes and wheelchair racing athletes. The age at peak performance varies according to sex, impairment type and event and increases gradually from sprint to endurance events. The best Top 100 performances include a large age range suggesting that performance has probably not been optimized yet for most elite para-athletes and wheelchair racers. The next Paralympic Games of Tokyo 2020 and Paris 2024 will certainly offer exceptional performance.

## Introduction

There are a number of unanswered questions relating to the components of performance in Paralympic sport. For example, how age affects para–athletic performance for different types of impairment? Is there an age difference at peak performance between para-athletes and able-bodied athletes? At the age of 19 after his gold medal, the blade runner Jonnie Peacock said at the Paralympic Games of London in 2012:” I lost my leg aged at 5… Now I’m 1.9 s behind Usain Bolt.” This comment questions the physical capacities of the para-athletes and the technological contribution of prostheses on the evolution of performance (Jones and Wilson, 2009; Weyand et al., 2009; Willick and Lexell, 2014; Dyer, 2015a; Baker, 2016). Since the first 1960 Paralympic Games, an increasing number of athletes with physical, visual or intellectual impairments have participated to elite para-athletic competitions (Dyer, 2015b; Fagher et al., 2016). Scientific and technological progress, such as prosthetic equipment, contributed to para-athletic promotion and the improvement of their performances (Lepers et al., 2012; Dyer, 2015b; Grobler et al., 2015). Indeed, beyond the impairment type, para-athletic performance is a complex process including both intrinsic parameters such as genetics, morphology (height and mass) or age and extrinsic factors such as environmental conditions (temperature, humidity, pollution), training methods, nutrition or technology (Berthelot et al., 2015; Blauwet et al., 2016).

In this framework, age is a major determinant of the performance in able-bodied athletes. Previous studies investigated the effect of age for different maximal physical performances (Moore, 1975; Baker and Tang, 2010; Guillaume et al., 2011; Berthelot et al., 2012; Allen and Hopkins, 2015; Marck et al., 2017b; Lepers et al., 2018; Marc et al., 2018) and showed a similar age-related pattern for maximal physical performances in Track and Field, swimming disciplines, or in tennis performance for high level athletes (Guillaume et al., 2011; Berthelot et al., 2012; Marck et al., 2017b). Maximal performance gradually increases with age until it reached a peak around 25–30 years according to the type of sport event. Then, it exponentially declines due to the aging process (Marck et al., 2017b). All the physiological systems involved in locomotion are intimately linked in the process of development and aging. During childhood and adolescence, there is an increase in muscle mass and strength. After maturity, skeletal muscle aging is characterized by a progressive loss of muscle mass. It is estimated that muscle mass losses are in the range of 0.02 to 3.3% per year (Mitchell et al., 2012; McGregor et al., 2014). These alterations of muscle with age remain intimately related to the degradation of the other systems with which it constantly interacts (Mitchell et al., 2012). This loss of mass can vary greatly from one muscle to another and the muscle mass loss in the legs may be higher compared to the arms (Lepers et al., 2012; Mitchell et al., 2012). This could impact the slope of performance decline that illustrates athlete’s impairment.

In para-athletics, no study has reported age related performance determinants. They only focused on technology (Weyand et al., 2009; Cooper and De Luigi, 2014), biomechanical analyses (Frossard, 2012; Beck et al., 2016), or incidence of injuries (Gawronski et al., 2013; McNamee et al., 2014; Fagher et al., 2016). Furthermore, the performance gap between able-bodied athletes and para-athletes, though well perceived in the daily life, has not been precisely quantified in all Track and Field events.

The present study characterizes the age-related changes in maximal performance and estimates the peak age for 47 para-athletics and wheelchair racing events, with a total of 472 event classes (that considers all impairment types) for both female and male para-athletes. These are scaled to the all-time able-bodied record performance. We also compare the patterns of performance decline for the para-athletes and wheelchair racing athletes and determine the classes contributing to the maximal performance of each event.

## Materials and Methods

### Data Collection

Data (53,554) including athlete’s best performance of the year, event, age, and disability classification were collected for all International Paralympic Committee (IPC^1^) competitions from 2009 to 2017 for 47 female and male para-athletes and wheelchair events (see details in Table 1) incorporating a total of 472 sport classes. Before 2009, data were unavailable online. These performances were achieved by 7,231 athletes: 3,500 male para-athletes, 1,348 female para-athletes, 1,650 male wheelchair athletes, and 733 female wheelchair athletes.

**Table 1:** Number of performances and age indicators by event for para-athletes (PA) and wheelchair racing athletes (WCA) included in the database.

Event/Categories	Para-athletes male	Para-athletes female	Wheelchair athletes male	Wheelchair athletes female
100 m	3885	2115	1872	810
200 m	3271	1725	1690	723
400 m	2529	1003	1981	799
800 m	1299	224	1553	654
1 500 m	1458	320	1169	458
5 000 m	618	65	634	211
10 000 m	164	/	109	/
Marathon	319	50	601	174
Discus	2038	936	1969	935
Shot put	2256	1150	2086	1135
Javelin	1639	671	1550	856
Long jump	2110	1061	/	/
Triple jump	282	10	/	/
High jump	387	/	/	/


For the able-bodied maximal performance, the all-time world records by event at the end of 2017 were collected on the International Association of Athletics Federation’s website^2^.

### Study Design

For running events, racing times were converted to average speed in meters per second (ms^-1^). All performances were analyzed according to age (in years), speed (in ms^-1^) or meters (m) for throwing and jumping events and classes of disability. For males and females in all events, a unique maximal performance was selected among all individuals for each age. To compare the age-related pattern in performance between para-athletes and wheelchair racing athletes, two main categories were allocated: PA (Para-athletes) and WCA (Wheelchair athletes). Impairments types were regrouped according to IPC competition classification. For PA, these were; athletes with visual impairment (VI): T11-T12-T13-F11-F12-F13, athletes with cerebral palsy (CP): T35-T36-T37-T38-F35-F36-F37-F38, athletes with upper limb disabilities (UL): T45-T46-T47-F45-F46-F47, athletes with lower limb disabilities (LL): T42-T43-T44-F42-F43-F44 and athletes with intellectual disabilities (ID): T20-F20. In addition, for WCA, the classifications were: athletes with cerebral palsy in a wheelchair (CPW) T33-T34-F31-F32-F33-F34, athletes with tetraplegia disabilities (TD): T51-T52-F51-F52 and athletes with paraplegia disabilities (PD) T53-T54-F53-F54-F55-F56-F57-F58.

### Characterization of the Age-Performance Relationship

To characterize age-related changes in maximal performance, the data were fitted with the Moore equation, which is a double exponential function (simple inverted U-shaped) initially developed on the athletes running speed-age relationship (Moore, 1975).

$$Eqn1:P(t)=a(1-e^{-bt})+c(1-e^{dt})\;with\;a,\;b,\;c,\;d>0$$

P(t) is the performance (t the time), a and c are scaling parameters, b and d are the characteristic times of the exponential growth and decline, respectively. For an estimated performance P, the model can be described as the sum of two von Bertalanffy’s growth functions (VBGF): P(t) = A(t) + B(t) where A(t) is the increasing exponential process (first VBGF) and B(t) the decreasing exponential process (the second VBGF is modified with *d* > 0). The Moore equation (Eqn 1) allows the estimation of the age (in years) at peak performance. These coefficients are determined using a least-square non-linear regression (Marck et al., 2017b). The quality of each fit was estimated by the coefficient of determination R^2^ and the Root Mean Square Error (RMSE) (see Supplementary Table S1).

To quantify the gap between PA, WCA and able-bodied athletes, all datasets were scaled by maximal able-bodied performance of the event using the following formula:

Eqn2:Scaledperformance=performance/maximalable−bodiedperformance of the event.

For each event, the exact peak age was computed and corresponded to the age when the performance was maximal. All performances [speed (m/s) or meter (m)] were reported in percentages (%) in order to compare the events.

### Distribution of Age

In a complementary approach of the characterization of the age-related performance patterns, this study represented the age of the 100 best performances for PA and WCA by event using the heat map function of Matlab software. This visualization enhances a better visibility of the peak and range of athlete distribution using a density scale [scale: 0 (low density) to 15 (high density)].

### Distribution by Class for Best Performances

To assess the impact of each impairment type to the best performance, the number of sporting classifications within the top 100 best performances of each event were calculated as a percentage (%) for each sex.

All analyses were performed using the Matlab (MathWorks Inc.,) 2017b 9.3.0 software.

### Ethics Statement

This study was designed and monitored by the IRMES (Institut de Recherche bio-Médicale et d’Epidémiologie du Sport) scientific committee. It used a research protocol qualified as non-interventional, in which “…all acts are performed in a normal manner, without any supplemental or unusual procedure of diagnosis or monitoring.” (Article L1121–1 of the French Public Health Code).

## Results

### Age at Peak Performance

**FIGURE 1:**
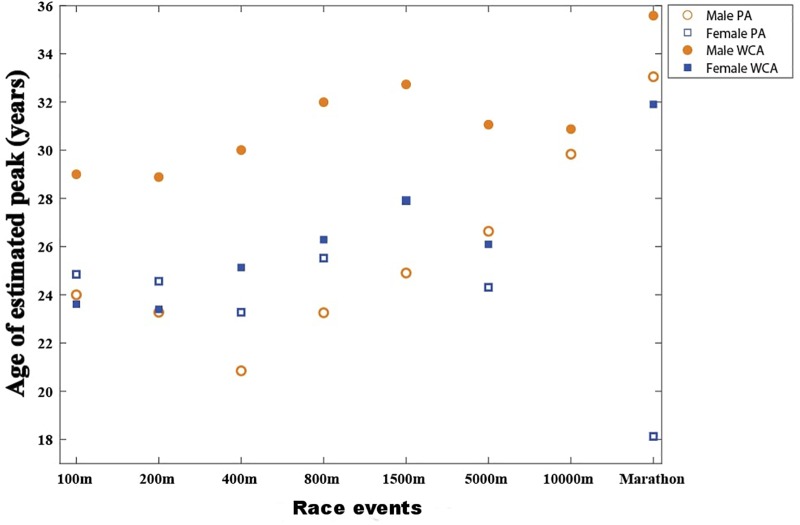
Age of estimated peak performance with the Moore equation in race events for male para-athletes (PA: 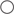) and male wheelchair racing athletes (WCA: 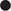); for female para athletes (PA: 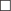) and female wheelchair racing athletes (WCA: 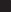).

**FIGURE 2:**
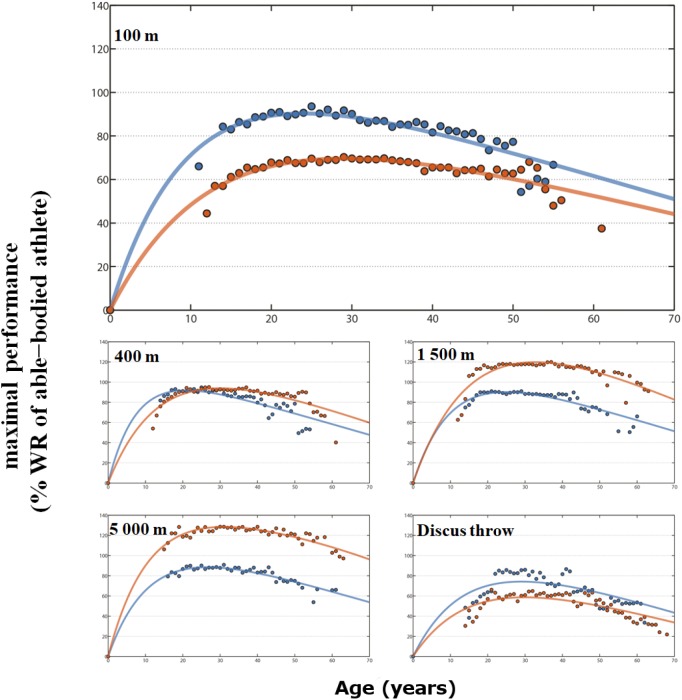
Envelope curve in % of maximal able-bodied performance for male para-athletes (blue line) and wheelchair racing athletes (red line) by age on 100 m, 400 m, 1500 m, 5000 m and discus throw. Peak age and R^2^ in Supplementary Table S1.

**FIGURE 3:**
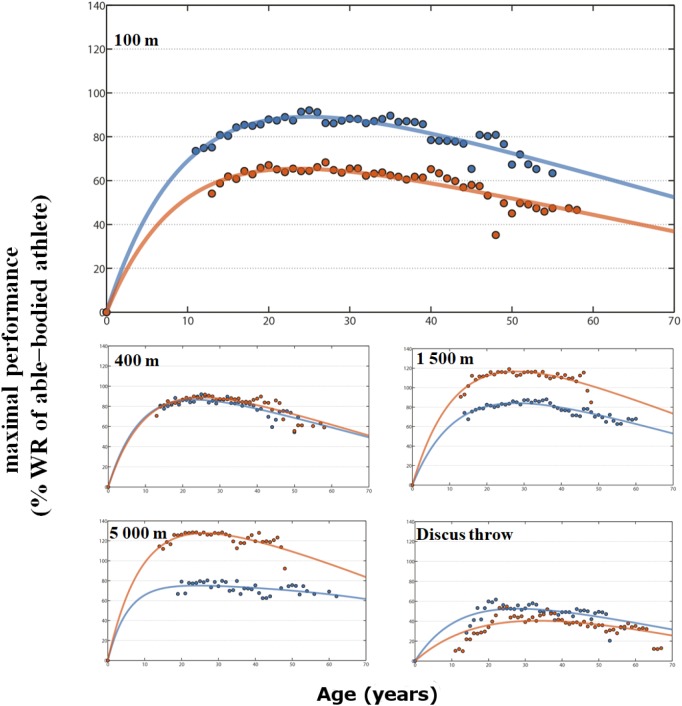
Envelope curve in % of maximal able-bodied performance for female para-athletes (blue line) and wheelchair racing athletes (red line) by age on 100m, 400m, 1500m, 5000m and discus throw. Peak age and R^2^ in Supplementary Table S1.

In all Track and Field events, the age-performance relationship showed a similar pattern for both PA and WCA categories in female and male athletes. There was a gradual progression of the best performances up to a peak and thereafter performance progressively declined. The age of the estimated peak performance varied according to the event (Figure 1–3; details in Supplementary Table S1).

In male PA sprinting events, the age of the estimated peak ranged from 24.0 years in the 100 m to 20.8 years in the 400 m (Figure 1, 2). In endurance events, the estimated peak was 23.2 years for the 800 m and 33.0 years for the marathon (Figure 1). In throwing events, the age of the estimated peak varied from 24.1 years for javelin to 29.3 years for shot-put; in jumping events from 23.2 for triple jump to 26.0 years for high jump. In male WCA events, the estimated peak occurred later and ranged from 29.0 years in the 100 m to 35.6 years for the marathon (Figure 1, 2).

In female PA sprinting events, the estimated peak ranged from 24.8 years in the 100 m to 23.3 years in the 400 m (Figure 1, 3). In endurance events, no relation occurred between the age of the estimated peak performance and distance. In the marathon, the estimated peak was 18.1 years (Figure 1). In throwing events, the age of the estimated peak varied from 26.9 years for javelin to 33.4 years for shot-put; in jumping events from 22.8 for triple jump to 26.4 years for long jump.

Similar to male, the peak performance among female WCA ranged from 23.6 years in the 100 m to 31.9 years for the marathon (Figure 1, 3). In throwing events, the study showed a later peak compared to PA, with an estimated peak of 33.6 years for discus and 38.3 years for shot-put.

### Performance Differences

In sprinting events, both female and male PA were faster than WCA. The difference decreased with distance and maximal performances were similar in the 400 m track event. For both sexes, maximal performances were lower than able-bodied best performances. In endurance events, PA were slower than WCA with the gap increasing as distance increased. In comparison to the best able-bodied athletes, WCA were faster (from +10.3% for female and +10.7% for male in the 800 m to +53.4% and +57.5% in the marathon) while PA were slower. In all throwing events, both female and male PA reached better performances than WCA, though all maximal performances were lower than able-bodied best performances (from -14% for male PA discus to -58% for female wheelchair javelin) (see details in Supplementary Table S1).

### Age-Range for Optimal Performance

**FIGURE 4:**
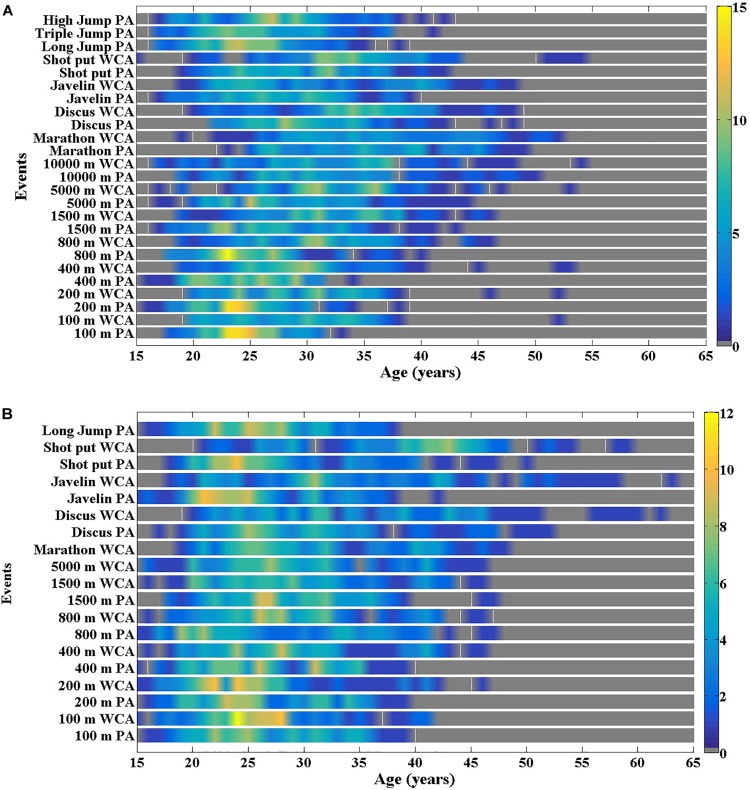
Density distribution of the 100 best performances by age for male **(A)** and female **(B)** for para-athletes (PA) and wheelchair racing athletes (WCA).

For both sexes, the age-range of the top 100 best performances was determined (Figure 4A,B). The findings showed a widespread age distribution from 20 to 60 years old and indicated that the density of performance was maximal around a peak age between 20 to 30 years old dependent upon event and sexes.

### Class of Disabilities and Optimal Performance

**FIGURE 5:**
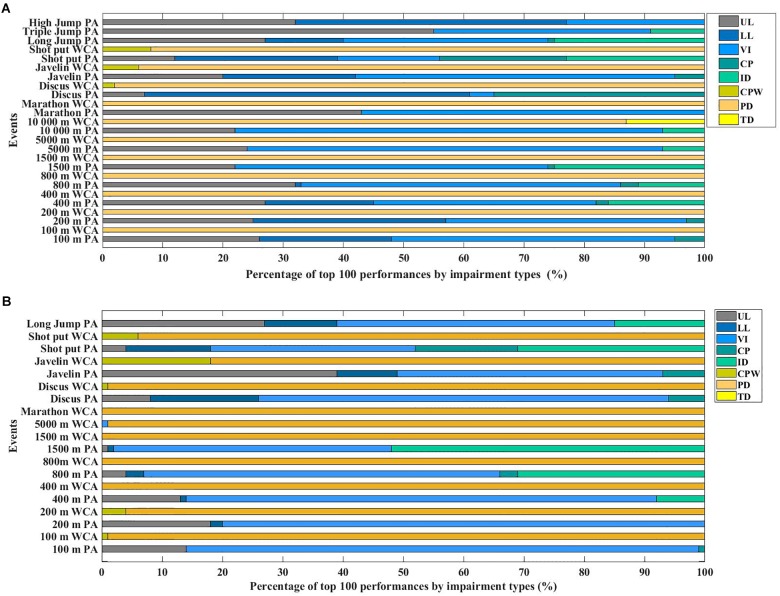
Percentage of impairment types in the 100 best performances for male **(A)** and female **(B)** for PA and WCA.

All classes of disabilities were represented within best PA performances (Figure 5A,B). In WCA events for both sexes, the vast majority of the top 100 performances were achieved by athletes from the paraplegia impairments types.

Among female PA, athletes with VI were represented in the top 100 for all events. They represented 78% (400 m) to 85% (100 m) of top 100 best results in sprinting events and 34% for the shot-put events. Athletes with an ID were mostly represented in middle-distance events (31% for the 800 m; 52% for the 1500 m) and shot-put. Athletes with UL were mostly represented in sprinting events (13% in the 400 m; 18% in the 200 m), in javelin (39%) and long jump (27%). Athletes with a LL in long-jump (12%) and throwing events (10% in javelin; 18% in discus. Finally, CP were represented in throwing events (6% in discus; 17% in shot-put) and mostly absent from the other events.

The male distribution of the top 100 was similar to female distribution. CP athletes were represented by 5% in javelin and 35% in discus but mostly absent from the other events. Similarly, athletes with a LL were 22% in javelin and 54% in discus, 13% in long jump, 45% in high jump, 32% in the 200 m, and 18% in the 400 m. UL athletes were represented in all events (7% in discus; 55% in triple jump). Athletes with an ID were mostly represented in middle- and long-distance running events (7% in the 10,000 m; 25% in the 1500m), in shot-put (23%), long jump (25%) and triple jump (9%). Male VI athletes were represented in the top 100 of all events (up to 71% in the 10,000 m).

## Discussion

### The Age at Maximal Peak Performance

Age-related changes in maximal performances for both PA and WCA revealed a similar pattern. The age at peak performance varied depending on sex, impairment classification and event. Such an age difference may depend on the “mosaic” of aging processes that do not homogenously alter the organism but that similarly impacts each type of impairment.

The collected data on 53,554 PA and WCA performances showed a similar pattern of progression in the performance-age relationship for all events, with an initial gradual increase in performance until reaching the peak, then a gradual decrease in maximum performance with age. The maximal PA and WCA performances fitted with the Moore equation showed that the respective determination coefficients (R^2^) of this equation were well adjusted to the maximum age-related performances (Figure 2, 3). Following this similar age-related performance pattern illustrated in many sport events and different species (Guillaume et al., 2011; Berthelot et al., 2012; Marck et al., 2017b; Marc et al., 2018), the para-athlete age-related performances confirmed the reliability and the robustness of the Moore equation (Eqn 1).

In male sprinting events (Figure 1, 2), PA peak age was similar to able-bodied athletes and increases in endurance events (Moore, 1975; Berthelot et al., 2012; Marck et al., 2017b; Marc et al., 2018). It is well known that the aging process involves physiological and psychological changes, whether structural or functional (Mitchell et al., 2012; McGregor et al., 2014). The number of muscle cells and of motor units decreases sharply with age (Faulkner et al., 2007). However, fast type II fibers (favored in sprint races) are more prematurely altered than slow type I fibers (favored in endurance racing). The increase in peak performance with the event duration and distance could therefore be an explanation for PA male results. For female PA (Figure 1, 3), the appearance of a plateau and a young age for marathon peak may be mainly related to a low number of participants (50 archived performances only, vs. 319 in male PA marathon or 2115 in women PA 100 m).

Furthermore, at the individual scale, the origin and development of the disability, either born with it or acquired later in life, is also an element in the age at peak performance or the appearance of not clear peak, which needs to be investigated. Indeed, PA seem to start their career younger due to the innate origin of the disability (Ravensbergen et al., 2018), while WCA begin their career and peak at a later age because of an impairment acquired during the adolescence or the young adult age (Ravensbergen et al., 2018). Depending upon the disability or the technical equipment, an adaptation period, in order to acquire an optimized techno-physiological interaction, may contribute to an older age at peak performance.

In some events, WCA age-related performance curves did not show a discernible peak performance. Power of the upper arms, muscle strength of the elbow extensors, muscle endurance of brachial triceps (Mitchell et al., 2012) and push angle (Lepers et al., 2014) may contribute to this observation. Such an element certainly increases the inter-individual variability for the age at peak performance.

The Top 100 performances included a large age range from 15 to 55 years (Figure 4) revealing that performance is not yet optimized for most of the elite PA and WCA. For the best male and female able-bodied athletes, the age range varies from 21 to 36 years for the 100 m to 20 to 38 years for the marathon (Marc et al., 2018), which indicates a tighter age-performance range. These results could be explained by the fact that able-bodied athletes who have competed since the first Olympic Games in 1896 represent an accumulated pool of 30,535 able-bodied athletes over 100 years including the 2016 Rio Olympic Games by comparison, the first Paralympic Games held in 1960 only giving 56 years of data on 12,752 para-athletes. Therefore, these lower numbers of participants can also be attributed to disability-induced barriers to mobility. Para-athletes may experience difficult interactions with their social and physical environments which leads to limitations in their activities of daily life, and restrictions in competition participation. Depending on the degree of functional loss, the para-athletes are impacted differently by contextual, environmental and personal factors (Fellinghauer et al., 2012). In addition, such a large age-band might be typical of para-athletes and reflect the performance of those born without impairment who often are athletes with accidental or progressive impairments appearing during their life.

For the best performances, only a few classes contributed to the maximal performance (Figure 5). For WCA, in both male and female events, the fastest athletes were in the PD class. Among paraplegic people, muscle strength in the upper extremities and respiratory function are comparable to that of the able-bodied population (Haisma et al., 2006a). In tetraplegic people, muscle strength varies greatly and respiratory function is considerably reduced relative to the values in an able-bodied population (Haisma et al., 2006b). This provides support for the lack of representation from this group in the 100 best performances achieved by the WCA. Among PA, the distribution seemed more heterogeneous. Overall, from 100 to 400 m, only male PA with a VI, LL and UL represented the best performances, whereas the female PA were predominantly in the VI category. Females with LL were less represented in sprint events. This could be explained by the fact that the mechanical properties of carbon prostheses may not be adapted yet to female specificities (such as developed strength or anthropometric factors – height or BMI (Sedeaud et al., 2014). With a constant stiffness, the lower strength produced by female PA could impact the prosthesis reaction, which depends on the magnitude of the applied force (Beck et al., 2016) thereby reducing the generated speed.

### The Performance Levels

Large differences remain between PA, WCA and able-bodied athlete’s performances, related to biomechanical properties, difference in equipment and sample size. In male and female sprint events, the best PA performances were 5 to 9% under the able-bodied world record. When race distance increases, so does this gap with a larger difference in females.

The PA’s maximal performances have come closer to the able-bodied world records but do not surpass them (Grobler et al., 2015). Nevertheless, maximal performances of able-bodied athletes have been plateauing for three decades and now seem to have reached their upper limits (Marck et al., 2017a). Similarly, the rate of progression of WCA seems to have considerably slowed down, at least through the observation of world records or the best performances such as in the Oita marathon (Lepers et al., 2012). WCA gap gradually increased with the race distance. The best WCA performances from 800 m to the marathon were, respectively, 10 to 57% over the able-bodied world record. These increases demonstrate that WCA in endurance events are comparable to hand cycling events where technological and strategic contributions are different compared to able-bodied athletes (Lepers et al., 2014). Indeed, WCA are able to coast for recovery or energy conservation (Cooper and De Luigi, 2014), whereas able bodied runners must keep expending precious energy even during downhill sections.

Technological advances will undoubtedly increase the performance levels and at the same time improve the quality of the wheelchair or prosthesis use in the daily life. However, the environment and economic situation could be less favorable to the improvement of such innovations and may even play a crucial role in the regression of maximal performances in both able-bodied and disabled athletes due to travel, physical and financial difficulties. In this context, it would be important to continue to develop policies that increase and promote physical activity and sport for the beneficial effects on health, such as a decreased risk of chronic diseases and an improved quality of life (Global Recommendations on Physical Activity for Health, 2010), particularly for people with physical disabilities (de Hollander and Proper, 2018).

## Conclusion

Para-athletes and wheelchair athletes display an age-related pattern in maximal performances, similar to able-bodied athletes. The age at peak performance increases gradually from sprinting to endurance events for para-athletes. The Top 100 best performances include a large age range suggesting that performance has probably not yet been optimized for most elite para-athletes and wheelchair racers. The Paralympic Games of Tokyo 2020 and Paris 2024 will certainly offer exceptional performances which can still be improved upon for most of the elite wheelchair racing and para-athletes. Further studies will contribute to increasing knowledge about age-related changes and the origin of the impairment in para-athletes and wheelchair racing athletes.

## Data Availability

Publicly available datasets were analyzed in this study. This data can be found here: www.paralympic.org.

## Author Contributions

JS, PG, AnM, J-FT, AS, and AdM designed the research. JS, PG, AnM, AS, and AdM performed and analyzed the research. JS, PG, AnM, JA, J-FT, AS, and AdM wrote the manuscript. All authors read and approved the final manuscript.

## Conflict of Interest Statement

The authors declare that the research was conducted in the absence of any commercial or financial relationships that could be construed as a potential conflict of interest.

## Footnotes

1 www.paralympic.org

2 www.iaaf.org

## Supplementary Material

TABLE S1 Coefficients values, fitting indicators and age at the peak performance for all track and field events.

## References

[B1] Allen S. V. Hopkins W. G. (2015). Age of peak competitive performance of elite athletes: a systematic review. *Sports Med.* 45 1431–1441. 10.1007/s40279-015-0354-3 26088954

[B2] Baker A. B. Tang Y. Q. (2010). Aging performance for masters records in athletics, swimming, rowing, cycling, triathlon, and weightlifting. *Exp. Aging Res.* 36 453–477. 10.1080/0361073X.2010.507433 20845122

[B3] Baker D. A. (2016). The “Second Place” problem: assistive technology in sports and (Re) constructing normal. *Sci. Eng. Ethics* 22 93–110. 10.1007/s11948-015-9629-1 25649071

[B4] Beck O. N. Taboga P. Grabowski A. M. (2016). Characterizing the mechanical properties of running-specific prostheses. *PLoS One* 11:e0168298. 10.1371/journal.pone.0168298 27973573 PMC5156386

[B5] Berthelot G. Len S. Hellard P. Tafflet M. Guillaume M. Vollmer J.-C. (2012). Exponential growth combined with exponential decline explains lifetime performance evolution in individual and human species. *Age* 34 1001–1009. 10.1007/s11357-011-9274-9 21695422 PMC3682058

[B6] Berthelot G. Sedeaud A. Marck A. Antero-Jacquemin J. Schipman J. Saulière G. (2015). Has athletic performance reached its peak? *Sports Med.* 45 1263–1271. 10.1007/s40279-015-0347-2 26094000 PMC4536275

[B7] Blauwet C. A. Lexell J. Derman W. (2016). “Paralympic sports medicine,” in *Training and Coaching the Paralympic Athlete*, eds Vanlandewijck Y. C. Thompson W. R. (Chichester: John Wiley & Sons, Ltd), 75–95. 10.1002/9781119045144.ch4

[B8] Cooper R. A. De Luigi A. J. (2014). Adaptive sports technology and biomechanics: wheelchairs. *PM R* 6 S31–S39. 10.1016/j.pmrj.2014.05.020 25134750

[B9] de Hollander E. L. Proper K. I. (2018). Physical activity levels of adults with various physical disabilities. *Prev. Med. Rep.* 10 370–376. 10.1016/j.pmedr.2018.04.017 29755934 PMC5944414

[B10] Dyer B. (2015a). The controversy of sports technology: a systematic review. *Springerplus* 4:524. 10.1186/s40064-015-1331-x 26405644 PMC4575312

[B11] Dyer B. (2015b). The progression of male 100 m sprinting with a lower-limb amputation 1976–2012. *Sports* 3 30–39. 10.3390/sports3010030

[B12] Fagher K. Jacobsson J. Timpka T. Dahlström Ö Lexell J. (2016). The Sports-Related Injuries and Illnesses in Paralympic Sport Study (SRIIPSS): a study protocol for a prospective longitudinal study. *BMC Sports Sci. Med. Rehabil.* 8:28. 10.1186/s13102-016-0053-x 27579170 PMC5004301

[B13] Faulkner J. A. Larkin L. M. Claflin D. R. Brooks S. V. (2007). Age-related changes in the structure and function of skeletal muscles. *Clin. Exp. Pharmacol. Physiol.* 34 1091–1096. 10.1111/j.1440-1681.2007.04752.x 17880359

[B14] Fellinghauer B. Reinhardt J. D. Stucki G. Bickenbach J. (2012). Explaining the disability paradox: a cross-sectional analysis of the Swiss general population. *BMC Public Health* 12:655. 10.1186/1471-2458-12-655 22894722 PMC3528470

[B15] Frossard L. (2012). Biomechanical analyses of the performance of Paralympians: from foundation to elite level. Interview by Sarah A. Curran. *Prosthet. Orthot. Int.* 36 380–395. 10.1177/0309364612453257 22918919

[B16] Gawronski W. Sobiecka J. Malesza J. (2013). Fit and healthy Paralympians–medical care guidelines for disabled athletes: a study of the injuries and illnesses incurred by the Polish Paralympic team in Beijing 2008 and London 2012. *Br. J. Sports Med.* 47 844–849. 10.1136/bjsports-2013-092298 23902777

[B17] Global Recommendations on Physical Activity for Health (2010). *Global Recommendations on Physical Activity for Health.* Available at: http://www.ncbi.nlm.nih.gov/books/NBK305057/ [accessed December 18, 2018].

[B18] Grobler L. Ferreira S. Terblanche E. (2015). Paralympic sprint performance between 1992 and 2012. *Int. J. Sports Physiol. Perform.* 10 1052–1054. 10.1123/ijspp.2014-0560 25710327

[B19] Guillaume M. Len S. Tafflet M. Quinquis L. Montalvan B. Schaal K. (2011). Success and decline: top 10 tennis players follow a biphasic course. *Med. Sci. Sports Exerc.* 43 2148–2154. 10.1249/MSS.0b013e31821eb533 21502889

[B20] Haisma J. A. van der Woude L. H. V. Stam H. J. Bergen M. P. Sluis T. A. R. Bussmann J. B. J. (2006a). Physical capacity in wheelchair-dependent persons with a spinal cord injury: a critical review of the literature. *Spinal Cord* 44 642–652. 10.1038/sj.sc.3101915 16534502

[B21] Haisma J. A. van der Woude L. H. V. Stam H. J. Bergen M. P. Sluis T. A. R. Bussmann J. B. J. (2006b). Physical capacity in wheelchair-dependent persons with a spinal cord injury: a critical review of the literature. *Spinal Cord* 44 642–652. 10.1038/sj.sc.3101915 16534502

[B22] Jones C. Wilson C. (2009). Defining advantage and athletic performance: the case of Oscar Pistorius. *Eur. J. Sport Sci.* 9 125–131. 10.1080/17461390802635483

[B23] Lepers R. Stapley P. J. Cattagni T. (2018). Variation of age-related changes in endurance performance between modes of locomotion in men: an analysis of master world records. *Int. J. Sports Physiol. Perform.* 13 394–397. 10.1123/ijspp.2017-0222 28714746

[B24] Lepers R. Stapley P. J. Knechtle B. (2012). Gender differences in wheelchair marathon performance – Oita International Wheelchair Marathon from 1983 to 2011. *Open Access J. Sports Med.* 3 169–174. 10.2147/OAJSM.S37819 24198599 PMC3781911

[B25] Lepers R. Stapley P. J. Knechtle B. (2014). Analysis of marathon performances of disabled athletes. *Mov. Sport Sci.* 84 43–50. 10.1051/sm/2013078

[B26] Marc A. Sedeaud A. Schipman J. Saulière G. Toussaint J. F. (2018). Age and performance from 10 seconds to a 6-days race. *J. Athl. Enhanc.* 7:4 10.4172/2324-9080.1000298

[B27] Marck A. Antero J. Berthelot G. Saulière G. Jancovici J.-M. Masson-Delmotte V. (2017a). Are we reaching the limits of homo sapiens? *Front. Physiol.* 8:812 10.3389/fphys.2017.00812 PMC5662890 29123486

[B28] Marck A. Berthelot G. Foulonneau V. Marc A. Antero-Jacquemin J. Noirez P. (2017b). Age-Related Changes in Locomotor Performance Reveal a Similar Pattern for *Caenorhabditis elegans, Mus domesticus, Canis familiaris, Equus caballus*, and *Homo sapiens*. *J. Gerontol. A Biol. Sci. Med. Sci.* 72 455–463. 10.1093/gerona/glw136 27522057 PMC5861937

[B29] McGregor R. A. Cameron-Smith D. Poppitt S. D. (2014). It is not just muscle mass: a review of muscle quality, composition and metabolism during ageing as determinants of muscle function and mobility in later life. *Longev. Healthspan* 3:9. 10.1186/2046-2395-3-9 25520782 PMC4268803

[B30] McNamee M. Savulescu J. Willick S. (2014). Ethical considerations in paralympic sport: when are elective treatments allowable to improve sports performance? *PM R* 6 S66–S75. 10.1016/j.pmrj.2014.07.002 25134754

[B31] Mitchell W. K. Williams J. Atherton P. Larvin M. Lund J. Narici M. (2012). Sarcopenia, dynapenia, and the impact of advancing age on human skeletal muscle size and strength; a quantitative review. *Front. Physiol.* 3:260. 10.3389/fphys.2012.00260 22934016 PMC3429036

[B32] Moore D. H. II (1975). A study of age group track and field records to relate age and running speed. *Nature* 253 264–265. 10.1038/253264a0 1113841

[B33] Ravensbergen H. J. C. Genee A. D. Mann D. L. (2018). Expert consensus to guide the classification of paralympic swimmers with vision impairment: a delphi study. *Front. Psychol.* 9:1756. 10.3389/fpsyg.2018.01756 30386271 PMC6199393

[B34] Sedeaud A. Marc A. Marck A. Dor F. Schipman J. Dorsey M. (2014). BMI, a performance parameter for speed improvement. *PLoS One* 9:e90183. 10.1371/journal.pone.0090183 24587266 PMC3934974

[B35] Weyand P. G. Bundle M. W. McGowan C. P. Grabowski A. Brown M. B. Kram R. (2009). The fastest runner on artificial legs: different limbs, similar function? *J. Appl. Physiol.* 107 903–911. 10.1152/japplphysiol.00174.2009 19541739

[B36] Willick S. E. Lexell J. (2014). Paralympic sports medicine and sports science–introduction. *PM R* 6 S1–S3. 10.1016/j.pmrj.2014.05.022 25134746

